# An Apriori Algorithm-Based Association Rule Analysis to Identify Acupoint Combinations for Treating Diabetic Gastroparesis

**DOI:** 10.1155/2021/6649331

**Published:** 2021-03-25

**Authors:** Ping-Hsun Lu, Jui-Lin Keng, Fu-Ming Tsai, Po-Hsuan Lu, Chan-Yen Kuo

**Affiliations:** ^1^Department of Chinese Medicine, Taipei Tzu Chi Hospital, Buddhist Tzu Chi Medical Foundation, New Taipei City, Taiwan; ^2^School of Post-Baccalaureate Chinese Medicine, Tzu Chi University, Hualien, Taiwan; ^3^Department of Applied Mathematics, University of Washington, Seattle, WA, USA; ^4^Department of Research, Taipei Tzu Chi Hospital, Buddhist Tzu Chi Medical Foundation, New Taipei City, Taiwan; ^5^Department of Medicine, Mackay Medical College, New Taipei City, Taiwan; ^6^Department of Dermatology, Mackay Memorial Hospital, Taipei, Taiwan

## Abstract

We explored the potential association rules within acupoints in treating diabetic gastroparesis (DGP) using Apriori algorithm complemented with another partition-based algorithm, a frequent pattern growth algorithm. Apriori algorithm is a data mining-based analysis that is widely applied in various fields, such as business and medicine, to mine frequent patterns in datasets. To search for effective acupoint combinations in the treatment of DGP, we implemented Apriori algorithm to investigate the association rules of acupoints among 17 randomized controlled trials (RCTs). The acupoints were extracted from the 17 included RCTs. In total, 29 distinct acupoints were observed in the RCTs. The top 10 frequently selected acupoints were CV12, ST36, PC6, ST25, BL21, BL20, BL23, SP6, BL18, and ST21. The frequency pattern of acupoints achieved by using a frequent pattern growth algorithm also confirms the result. The results showed that the most associated rules were {BL23, BL18} ≥ {SP6}, {BL20, BL18} ≥ {PC6}, {PC6, BL18} ≥ {BL20}, and {SP6, BL18} ≥ {BL23} in the database. Acupoints, including BL23, BL18, SP6, BL20, and PC6, can be deemed as core elements of acupoint combinations for treating DGP.

## 1. Introduction

With the changes in lifestyle and the prevalence of obesity, International Diabetes Federation has reported a total of 451 million people with diabetes in the world, and the number of diabetes mellitus (DM) patients may rise to 693 million in 2045. DM is a non-negligible problem in developing countries [1]. Diabetic gastroparesis (DGP) is a common complication of autonomic neuropathy that occurs in patients with hyperglycemia or DM. Furthermore, DGP affects around 4.8% of patients with type I DM and 1% of those with type II DM [2]. Nausea, vomiting, early satiety, postprandial fullness, abdominal distension, and abdominal pain are often observed in patients with DGP. Wide glycemic fluctuations and impact on quality of life are also noticed in DGP patients [3, 4]. The possible pathogenesis of DGP includes multiple factors, such as vagus neuropathy, abnormal myenteric neurotransmission, damage of inhibitory neuronal function, and the impaired function of smooth muscle cells and interstitial Cajal cells (ICCs) [5–7]. The suggested treatment for DGP depends on the severity. For mild-to-moderate patients, recommendations consist of controlling blood sugar, adjusting diet, and using prokinetic agents. For severe patients, gastric electrical stimulation or placement of a feeding jejunostomy to provide nutrition is suggested. Gastrectomy is the last resort for DGP refractory to treatment [5]. Metoclopramide, erythromycin, domperidone, and cisapride are prokinetic agents that are commonly used to treat DGP, but they present short-term efficacy and may have adverse effects, including insomnia, extrapyramidal symptoms, and cardiac arrhythmias [5, 8]. To date, sufficient evidence that supports surgical treatment as beneficial for DGP is still lacking [8, 9]. Therefore, other alternative treatment options for DGP must be developed.

Acupuncture, an ancient therapy that has been used in China for over 2500 years, has become popular for its adjunctive role to ameliorate certain disease symptoms and to treat gastrointestinal tract disorders with limited side effects [10]. Acupuncture can improve gastric motility in animal models and humans [11, 12]. The possible mechanisms to improve the dyspeptic symptoms in DGP by acupuncture include restoration of the enteric nervous system (ENS), restoration of the ultrastructure and pacing function of ICCs, calibration of gastrointestinal hormone disorders, and improvement of gastrointestinal motility [13, 14]. The choice and combination of acupoints have been broadly accepted as important for successful acupuncture treatment. Yellow Emperor's Internal Classic and the ancient Meridian theory serve as guidelines for the selection of acupoints and their combination [15, 16]. Nevertheless, no consensus has been reached regarding the standard acupoint combinations for DGP treatment.

Data mining methods have been broadly applied in acupuncture. Data mining methods have been applied to evaluate the option and combination of acupuncture to treat reflux esophagitis [17], chronic atrophic gastritis [18], diarrhea [19], and diabetic peripheral neuropathy [20]. Given that acupuncture therapy focuses on numerous acupoints simultaneously in clinical practice, Apriori algorithm-based association rule analysis can be an essential and useful approach to explore the basic rules. Nevertheless, the acupoint combination for DGP treatment based on randomized controlled trials (RCTs) with a high level of evidence remains lacking. Data mining algorithms are frequently used in Chinese medicine and acupuncture combination analyses in the attempt to treat different types of syndromes [21]. Apriori, one of the association rule mining algorithms, proceeds by identifying the frequency of item sets in large databases and then determining their corresponding association rules [22]. Thus, Apriori algorithm provides comprehensive association rule analysis and offers insights into databases. Certain metrics, support, confidence, and lift are specified in Apriori to help us understand the strength of association rules [22]. Frequent pattern growth algorithm, on the other hand, mines the most frequent pattern and presents a dataset in the form of a tree with its structure constructed by the associations between items in the dataset. Similar to Apriori algorithm, it uses basic metrics, support, confidence, lift, and expected confidence to evaluate the frequent patterns [23].

In this paper, we primarily use Apriori algorithm to achieve the results, which are then further compared with the results obtained from the frequent pattern algorithm. We focused on the exploration of the promising core combination of acupoint combination to treat DGP by applying the Apriori association rule learning analysis based on RCTs [24].

## 2. Materials and Methods

### 2.1. Literature Search

Articles published up to January 2020 were searched for in PubMed, EMBASE, CENTRAL, and clinicaltrials.gov. The following MeSH and Emtree search headings were used: (diabet^*∗*^ AND gastroparesis) AND (acupuncture OR electroacupuncture OR acup^*∗*^). For different databases, we have adjusted the search terms accordingly. Unpublished studies were sought out from the ClinicalTrials.gov registry (http://clinicaltrials.gov/). Only human studies were included. We extracted data on the acupoints used for DGP treatment from these 17 RCTs, fourteen of which were included in a meta-analysis article (Table 1) [24–27].

### 2.2. Study Selection

We selected RCTs to assess the efficacy of acupuncture in the treatment of DGP patients. The following inclusion criteria were applied: randomized controlled trials with DGP diagnosis without gastric outlet ulceration or obstruction by radiographic examination, treatment including acupuncture, electroacupuncture (EA), abdomen acupuncture, eye acupuncture, ear acupuncture, scalp acupuncture, acupuncture combined with acupuncture-related auxiliary techniques and with the sham acupuncture, or gastroprokinetic agents as the control groups, and quantified data available for DGP symptoms. The exclusion criteria were the use of moxibustion, transcutaneous electroacupuncture, acupoint injection, acupuncture therapy combined with Chinese materia medica, incomplete data, and quantified data available for dyspeptic symptoms.

### 2.3. Risk of Bias (RoB) Assessment

Two authors independently assessed the methodological quality of the included studies using the Cochrane Collaboration's RoB tool [18]. Any disagreement between the investigators was resolved by the third author. Several domains were assessed, namely, allocation generation; allocation concealment; blinding of participants, personnel, and outcome assessors; completeness of outcome data; freedom from selective reporting; and freedom from other forms of bias.

### 2.4. Data Analysis

In this study, software RStudio (version 1.2.5033, Integrated Development for R, RStudio, PBC, Boston, MA) was used to conduct Apriori association rule learning analysis and plot charts [28]. The dataset contains 14 columns, where each column represents individual formulas. The dataset was then fitted by using the R package “arules.” Data visualization of the charts was performed by fitting the dataset into the R package, “arulesViz.” In contrast, we utilized Python programming language's open-source package (Python Software Foundation, Python Language Reference, version 3.7), “pyfpgrowth,” to conduct a frequent pattern growth algorithm [29].

Association rule learning algorithm is one of the widely used techniques to detect and analyze relations and useful information from transaction data. Numerous studies implemented association rule learning algorithms to identify medicine compatibility and to find interdependence within medical record data [22]. The association rule learning algorithm contains an antecedent and consequent set, both of which are a set of items.

Support and confidence factors are essential parameters in association rule learning. Support estimates the frequency of an acupoint appearing in the 14 formulas. On the other hand, confidence measures the frequency of acupoint A appearing in the formula, given that acupoint B appears simultaneously. Expected confidence is the number of formulas that include the consequent set of acupoints divided by the total number of formulas. Lift is the likelihood of an increase in the consequent given a particular antecedent. Namely, lift is the probability of the acupoint B appearing when acupoint A is present in a DGP formula given an expected confidence. It equals the confidence divided by expected confidence. During the exploration of the association rules, users need to test multiple combinations of minimum values for support and confidence factors to discover the significant association rules. However, the selection of thresholds showed slight ambiguity and varied from case to case. If the parameter thresholds were set at extremely high values, then certain meaningful information would be discarded.

In this study, the minimum thresholds for support and confidence factors were 20% and 60%, respectively. The highest value of the support factor was 0.6, and by increasing the minimum value of support from 0.1 to 0.2, we can filter out 82 rules down to 19 rules given that the confidence's minimum value was set to 0.6. The association rules were sorted by support factor in descending order.

## 3. Results

### 3.1. Study Characteristics

The acupuncture treatment for DGP used an average of five acupoints, and the average duration of treatment was 22 days. In the meta-analysis, compared with the control group, acupuncture therapy showed a higher response rate (RR, 1.20 (95% confidence interval (CI), 1.12 to 1.29), *P* < 0.00001) and significantly improved dyspeptic symptoms like stomach fullness, loss of appetite, and nausea and vomiting in the treatment group, but there is no real difference between the two groups of gastric emptying [24]. Li et al. reported that in patients with diabetic gastroparesis, acupuncture reduces gastric retention, improves gastroparesis symptoms [25], and is effective for epigastric fullness [27]. Song et al. showed acupuncture treatment improved the DGP symptom score and reduced serum content of transmembrane protein 16A (a selective marker of ICC) [26].

### 3.2. RoB Assessment

Table 1 summarizes the methodological quality of the included studies, whereas Supplementary Figure 1 provides the detailed RoB assessment. The 17 RCTs exhibited variable overall quality. This finding can be possibly explained by one of the following reasons. The random sequences of 11 studies out of 17 trials were correct and had apparent descriptions [24–27]. None of the retrieved studies gave a detailed description of allocation concealment, only two trials used patient blinding with sham electroacupuncture [24, 25], seven studies reported incomplete data, and seven studies did not give the baseline comparison of the severity of DGP symptoms [24].

### 3.3. Acupoint Distribution

**Figure 1:**
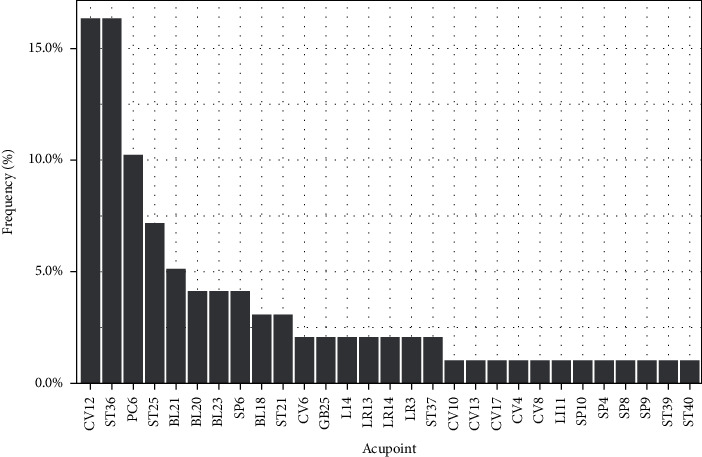
Frequency distribution of acupoints used in the 17 RCTs of acupuncture treatment for DGP.

We identified 29 acupoints from the 17 RCTs on acupuncture treatment for DGP.  Figure 1 shows the frequency distribution of acupoints. The 10 most frequently selected acupoints for treating DGP were CV12, ST36, PC6, ST25, BL21, BL20, BL23, SP6, BL18, and ST21.

### 3.4. Apriori Algorithm-Based Association Rule Analysis for Item Sets of Herb Combinations

**Figure 2:**
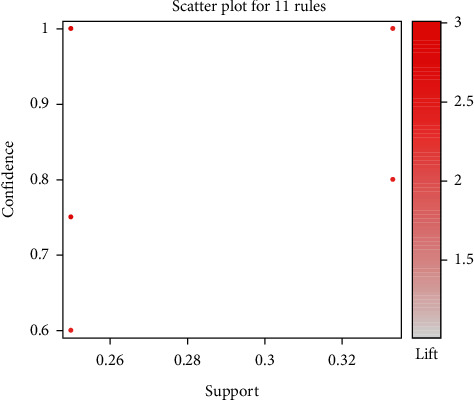
Scatter plot for the 11 association rules obtained in the 17 RCTs of acupuncture treatment for DGP via an Apriori algorithm-based association rule analysis.

We investigated 11 association rules in accordance with the 17 acupoint formulas for treating DGP. The association rules were shown based on the scatter plot with support on the *x*-axis and confidence on the *y*-axis. The color of each association rule was decided by the metric's corresponding value and lift (Figure 2). The results demonstrated that the lift values of 11 association rules were larger than 1. Thus, the likelihood of the antecedent and consequent acupoints being selected in the same formula was statistically significantly larger than that of consequent acupoint being selected alone. The metric values for the confidence of all 11 association rules were at least larger than 0.6. Therefore, for each rule, the likelihood of the consequent acupoint being selected when antecedent acupoints are selected is relatively high. By contrast, the metric values for support ranged from 0.2 to 0.6, indicating the frequency of each individual antecedent acupoint appearing in the formula. This finding suggests the presence of a core list of acupoints to select from for the treatment of DGP. All 11 association rules were ordered by the metric and support (Table 2).

**Figure 3:**
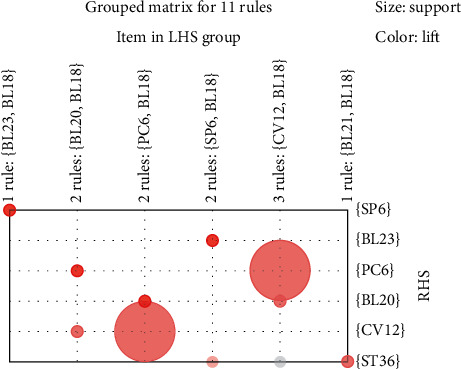
Grouping matrix for the 11 association rules obtained in the 17 RCTs of acupuncture treatment for DGP via an Apriori algorithm-based association rule analysis.

**Figure 4:**
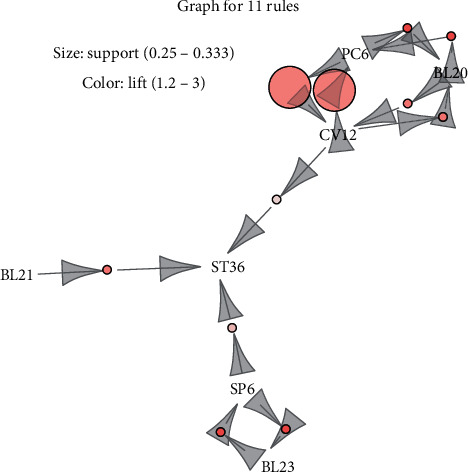
Combination matrix of association rules obtained in the 17 RCTs of acupuncture treatment for DGP via an Apriori algorithm-based association rule analysis.

A grouped matrix diagram was used to cluster similar rules into groups (circles) and display the general distribution of association rules (Figure 3). Six clusters were on the horizontal ordinate, and the vertical ordinate represented the acupoints generated by these six clusters (rules). The darker the color of a circle was, the higher was the degree of lift of a group. The size of a circle represents the degree of support. Thus, the larger the circle was, the higher was the degree of support.  Figure 4 shows the network graph visualization. The figure shows the 11 association rules found among the 17 acupoint formulas. Acupoints ST36 and CV12 were most likely to be used in association with other acupoints.

### 3.5. Apriori Algorithm-Based Association Rule Analysis for Item Sets of Herb Combinations Complemented with Frequent Pattern Growth Algorithm

Table 3 shows the frequent patterns of acupoint used in the 17 RCTs based on frequent pattern growth algorithm, and its results are coherent with the result in Figure 4. However, the values of lift to the association rules containing ST36 and CV12 were low, and thus they were not selected in the following result. Acupoints {BL23, BL18} ≥ {SP6}, {BL20, BL18} ≥ {PC6}, {PC6, BL18} ≥ {BL20}, and {SP6, BL18} ≥ {BL23} were selected, revealing item sets of antecedent and consequent acupoints. The results of selected association rules matched the association rule #8 {BL23} ≥ {SP6}, #3 {BL20} ≥ {PC6}, #4 {PC6} ≥ {BL20}, and #9 {SP6} ≥ {BL23} in Table 2. Likewise, the results of selected association rules are consistent with #17 (“PC6,” “SP6”) ≥ (“CV12,” “ST36”), #19 (CV12, SP6) ≥ (PC6, ST36), # 6 (BL18, CV12) ≥ (BL20, BL21, BL23), #9 (BL23, CV12) ≥ (BL20, BL21), #4 (BL20, BL21) ≥ (BL23, ST36), #5 (BL21, BL23) ≥ (BL20, ST36), #17 (PC6, SP6) ≥ (CV12, ST36), and #7 (BL21, CV12) ≥ (ST36) in Table 4.

## 4. Discussion

**Figure 5:**
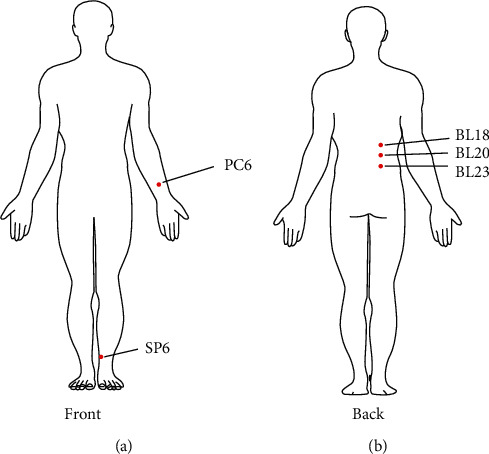
Location of core acupoints derived from association rules obtained in the 17 RCTs of acupuncture treatment for DGP.

This study indicated BL23, BL18, and SP6 and BL20, BL18, and PC6 as the major acupoint combinations in treating DGP, as confirmed by Apriori association-mining analysis (Figure 5). RCTs of acupuncture for DGP revealed that the acupoint combination played a role in relieving dyspeptic symptoms in patients with DGP.

The evidence-based strategies help to ascertain the efficacy of selecting acupoints for further treatment. To the best of our knowledge, this study is the first report on potential core acupoint combinations to treat patients with DGP based on RCTs and data mining analysis. The core acupoint combinations are helpful for patients with DGP; the possible fundamental mechanisms include restoration of ICC [13, 30], mediation of hormones, restoration of the ENS, and enhancement of gastrointestinal motility [14, 31]. Stimulating ST36 and CV12 points of the DGP rat with EA can reduce ICC apoptosis and restore ICC structure and pacing function, possibly through the mouse stem cell factor (SCF)/KIT-ETV1 signaling pathway to upregulate the mRNA of SCF and neuronal nitric oxide synthase [32–34]. Hou et al. reported an RCT that included 40 postoperative patients with gastrointestinal tumors; this RCT showed that electrical stimulation on acupoints ST36 and ST37 can elevate the serum levels of gastrin and motilin and electrogastrogram frequency. Additionally, the recovery of gastrointestinal function and reduction of complications, such as flatulence, abdominal pain, and diarrhea, were noted [35]. Jang et al. reported that stimulation with acupoint ST36 in mice can promote the motility of the small intestine via the decrease in serum levels of vasoactive intestinal peptide and an increase in serum levels of motilin, ghrelin, and gastrin [36]. Liang et al. demonstrated that electrical stimulation on acupoints ST36 and ST25 of a constipation mouse model can improve gastrointestinal motility by regulating excitatory and inhibitory neurons in ENS [37]. High-frequency electrical stimulation of ST36 in diabetic rats can promote the regeneration of lost enteric neurons through glial cell-derived neurotrophic factor and phosphatidylinositol-3 kinase/Akt signaling pathways [38].

The combination of acupoints was reported to exhibit greater therapeutic efficacy than a single acupoint [39, 40]. Wang et al. reported that the combination of multiple acupoints resulted in improved the score of the Pittsburgh sleep quality index and Athens Insomnia Scale via regulation of the activity of the brain area related to sleep experience [39]. The RCT which enrolled 30 patients with hypertension showed that the combined acupuncture points LR3 and KI3 can activate a wider area of the brain than a single point acupuncture based on the resting-state functional magnetic resonance imaging scan [41]. Gao et al. demonstrated that stimulating acupoint combination in slow transit constipation mice showed higher defecation grain number and intestinal propulsion rate compared with a single acupoint [42]. In DGP model rats, stimulation of the combined acupoints ST36 and CV12 can produce more ICCs than the single point ST36 [43]. Hence, an acupoint combination displays better pharmacological efficacy than a single acupoint. Even though there are also articles discussing the compatibility laws of acupuncture in the treatment of DGP, it uses fewer databases, not selects a higher level of evidence article, such as RCT, and does not elaborate on the association analysis [44, 45]. We implemented systemic review and Apriori algorithm to investigate the association rules of acupoints among 17 RCTs to demonstrate the effectiveness of acupuncture on patients with DGP. Moreover, research studies indicate that the core acupoint combinations we discovered can improve DGP through the protection of ICCs, amelioration of vascular endothelial dysfunction, hormone modulation, and stimulation of neurons [46–53] (Table 5).

### 4.1. Limitation

Despite our results on potential core acupoint combinations for the treatment of DGP, our study has several limitations. First, most RCTs included in this meta-analysis revealed a high RoB, and only two studies used the blinding method. Thus, a high RoB should be considered to explain these results. Second, most RCTs lacked a careful follow-up. The treatment duration was 22 days on average in this meta-analysis. Therefore, further research should be carried out regarding the long-term efficacy and safety of these core acupoint combinations for DGP. Third, as the mechanisms of action of acupoint combinations are unclear, more basic and clinical studies are required for a comprehensive assessment.

## 5. Conclusions

Based on RCTs and data mining analysis, the acupoint combinations of BL23, BL18, and SP6 and BL20, BL18, and PC6 are potential acupuncture treatments for DGP. The acupoint combinations also significantly improved stomach fullness, nausea and vomiting, and loss of appetite. However, further trials with larger sample sizes and appropriate controls are recommended.

## Figures and Tables

**Table 1:** Summary of 17 RCTs of acupuncture treatment for DGP.

Study (year)	Study design	Inclusion criteria	Acupoints	Overall bias
Wang et al. (2008)	RCT	DGP	ST36, LI4	High
Ge et al. (2010)	RCT	DGP	CV12, ST36, PC6	High
Shen et al. (2010)	RCT	DGP	PC6, CV12, CV6, ST36, SP6	High
Wang et al. (2010)	RCT	DGP	BL20, BL21, BL18, BL23, PC6, ST36, SP6, CV12	High
Zeng and Chai (2008)	RCT	DGP	BL21, CV12, BL20, LR13, BL23, BL18, LR14, GB25, ST25, ST36	High
Zhang et al. (2007)	RCT	DGP	CV12, ST36, PC6	High
Zheng and Ge (2010)	RCT	DGP	CV12, ST36, PC6	High
Han et al. (2001)	RCT	DGP	ST36, ST25, PC6, ST39, CV12	High
Li (2006)	RCT	DGP	ST36, CV12, ST25, BL21, BL20, LR3, BL23, PC6	High
Wang (2007)	RCT	DGP	BL21, CV12, BL20, LR13, BL23, BL18, LR14, GB25, ST25, ST36	High
Wang et al. (2009)	RCT	DGP	CV17, CV13, CV12, CV4, CV10, CV8, CV6	High
Chen (2008)	RCT	DGP	CV12, ST36, ST25, ST21, ST37	High
Zhao (2011)	RCT	DGP	ST36, CV12, ST25, ST21, ST37	High
Chen (2005)	RCT	DGP	CV12, ST21, ST25, BL21, ST36	High
Zhang et al. (2013)	RCT	DGP	CV12, ST36, PC6	High
Li et al. (2015)	RCT	DGP	CV12, ST36, PC6	Low
Song et al. (2020)	RCT	DGP	CV12, ST36, PC6, SP9, SP10, SP6, SP8, LI11, LI4, ST40, LR3, SP4	High

RCT: randomized controlled trial; DGP: diabetic gastroparesis.

**Table 2:** Apriori algorithm-based association rules for acupoints used in the 17 RCTs on acupuncture treatment for DGP.

No.	Association rules	Support	Confidence	Expected confidence	Lift
1	{PC6} ≥ {CV12}	0.3333333	1.0000000	0.4166667	2.400000
2	{CV12} ≥ {PC6}	0.3333333	0.8000000	0.4166667	2.400000
3	{BL20} ≥ {PC6}	0.2500000	1.0000000	0.3333333	3.000000
4	{PC6} ≥ {BL20}	0.2500000	0.7500000	0.3333333	3.000000
5	{BL20} ≥ {CV12}	0.2500000	1.0000000	0.4166667	2.400000
6	{CV12} ≥ {BL20}	0.2500000	0.6000000	0.4166667	2.400000
7	{BL21} ≥ {ST36}	0.2500000	1.0000000	0.5000000	2.000000
8	{BL23} ≥ {SP6}	0.2500000	1.0000000	0.3333333	3.000000
9	{SP6} ≥ {BL23}	0.2500000	0.7500000	0.3333333	3.000000
10	{SP6} ≥ {ST36}	0.2500000	0.7500000	0.6666667	1.500000
11	{CV12} ≥ {ST36}	0.2500000	0.6000000	0.8333333	1.200000

DGP: diabetic gastroparesis.

**Table 3:** Frequent patterns of acupoints used in the 17 RCTs on acupuncture treatment for DGP.

No.	Frequent pattern	Support
1	(“CV12,” “ST36”)	1.071429
2	(“CV12,” “PC6”)	0.714286
3	(“PC6,” “ST36”)	0.714286
4	(“CV12,” “ST25”)	0.500000
5	(“ST25,” “ST36”)	0.500000
6	(“BL21,” “ST36”)	0.357143
7	(“BL21,” “CV12”)	0.357143
8	(“BL23,” “ST36”)	0.285714
9	(“BL23,” “CV12”)	0.285714
10	(“BL21,” “BL23”)	0.285714
11	(“BL20,” ‘BL23”)	0.285714
12	(“BL20,” “ST36”)	0.285714
13	(“BL20,” “CV12”)	0.285714
14	(“BL20,” “BL21”)	0.285714
15	(“CV12,” “SP6”)	0.285714
16	(“SP6,” “ST36”)	0.285714
17	(“PC6,” “SP6”)	0.285714
18	(“CV12,” “ST21”)	0.214286
19	(“ST21,” “ST36”)	0.214286
20	(“ST21,” “ST25”)	0.214286
21	(“BL18,” “ST36”)	0.214286
22	(“BL18,” “CV12”)	0.214286
23	(“BL18,” “BL21”)	0.214286
24	(“BL18,” “BL20”)	0.214286
25	(“BL18,” “BL23”)	0.214286

DGP: diabetic gastroparesis.

**Table 4:** 24 frequent pattern growth algorithm-based association rules obtained in the 17 RCTs on acupuncture treatment for DGP.

No.	LHS	RHS	Confidence
1	(BL18, BL20)	(BL21, BL23, ST36)	1.00
2	(BL18, BL23)	(BL20, BL21, ST36)	1.00
3	(BL20, BL23)	(BL21, ST36)	1.00
4	(BL18, BL21)	(BL20, BL23, ST36)	1.00
5	(BL20, BL21)	(BL23, ST36)	1.00
6	(BL21, BL23)	(BL20, ST36)	1.00
7	(BL18, CV12)	(BL20, BL21, BL23)	1.00
8	(BL21, CV12)	(ST36)	0.60
9	(BL20, CV12)	(BL21, BL23)	1.00
10	(BL23, CV12)	(BL20, BL21)	1.00
11	(BL18, ST36)	(BL20, BL21, BL23)	1.00
12	(BL21, ST36)	(CV12)	0.60
13	(BL20, ST36)	(BL21, BL23)	1.00
14	(BL23, ST36)	(BL20, BL21)	1.00
15	(ST21, ST25)	(CV12)	1.00
16	(ST21, ST36)	(ST25)	1.00
17	(CV12, ST21)	(ST25)	1.00
18	(PC6, SP6)	(CV12, ST36)	0.75
19	(SP6, ST36)	(CV12, PC6)	0.75
20	(CV12, SP6)	(PC6, ST36)	0.75
21	(CV12, ST25)	(ST36)	1.00
22	(ST25, ST36)	(CV12)	1.00
23	(CV12, PC6)	(ST36)	1.00
24	(CV12, ST36)	(PC6)	0.66

DGP: diabetic gastroparesis.

**Table 5:** Potential efficacy of the core acupoints for DGP treatment.

Point	Chinese name	English name	Primary meridians	Efficacy
BL18 [49]	Ganshu	Liver locus	Bladder	Activation of CRH-like neurons; modulation of the expressions of CRH and GR in the PVN
BL20 [54]	Pishu	Spleen locus	Bladder	Suppresses acid secretion and increases *β*-EP and SS
BL23 [49]	Shenshu	Kidney locus	Bladder	Activation of CRH-like neurons; modulation of the expressions of CRH and GR in the PVN
SP6 [50–52]	Sanyinjiao	Crossroad at three Yin	Spleen	Protection of ICCs, amelioration of vascular endothelial dysfunction, and modulation of ghrelin
PC6 [54–56]	Neiguan	Inner pass	Pericardium	Suppresses acid secretion, increases *β*-EP and SS, modulates vagovagal neurocircuits, and decreases the number of double-labeled OT neurons and c-fos neurons

DGP: diabetic gastroparesis; CRH: corticotropin-releasing hormone; GR: glucocorticoid receptor; PVN: paraventricular nucleus of the hypothalamus; *β*-EP; beta-endorphin; SS: somatostatin; GD: gastric distention; ICCs: interstitial Cajal cells; OT: oxytocin.

## Data Availability

The data utilized to support the findings of this study are included within the article.

## Supplementary Materials

Supplementary Materials Supplementary Figure 1: RoB assessment of the retrieved RCTs.
