# Supplementary figures and images for: An Apriori Algorithm-Based Association Rule Analysis to Identify Acupoint Combinations for Treating Diabetic Gastroparesis
Source: Evid Based Complement Alternat Med. 2021 Mar 25;2021:6649331. doi: 10.1155/2021/6649331 (PMC8018850; doi:10.1155/2021/6649331)

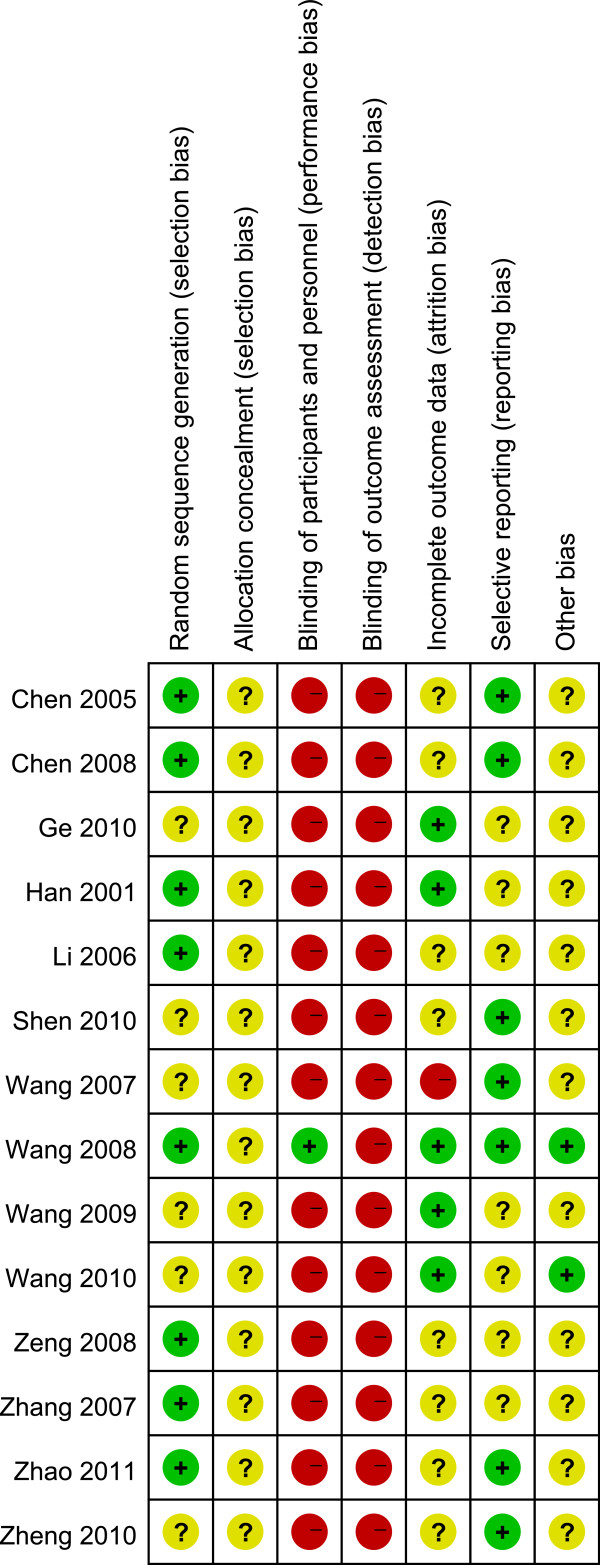

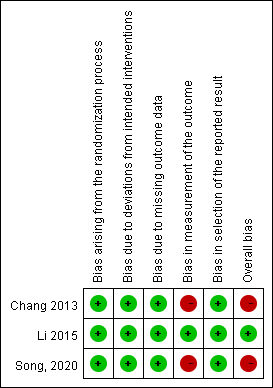


(a) (b)

Supplementary Figure 1. risk of bias assessment of the retrieved RCTs

Supplement: Supplementary Materials — Supplementary Figure 1: RoB assessment of the retrieved RCTs. [file 6649331.f1.docx]
